# The Role of Blood Clot in Guided Bone Regeneration: Biological Considerations and Clinical Applications with Titanium Foil

**DOI:** 10.3390/ma14216642

**Published:** 2021-11-04

**Authors:** Lucio Milillo, Fabrizio Cinone, Federico Lo Presti, Dorina Lauritano, Massimo Petruzzi

**Affiliations:** 1 Independent Researcher, 70126 Bari, Italy; lmilillo1@tin.it (L.M.); fabrizio.cinone@gmail.com (F.C.); 2 B.L. Consulting S.A.S.-Schio-Vicenza, 36015 Schio, Italy; federicolopresti.contact@gmail.com; 3 Centre of Neuroscience of Milan, Department of Medicine and Surgery, University of Milano-Bicocca, 20090 Monza, Italy; dorina.lauritano@unimib.it; 4 Department of Interdisciplinary Medicine, University of Bari “Aldo Moro”, 70126 Bari, Italy

**Keywords:** blood clot, titanium foil, bone augmentation, guided bone regeneration

## Abstract

In Guided Bone Regeneration (GBR) materials and techniques are essential to achieve the expected results. Thanks to their properties, blood clots induce bone healing, maturation, differentiation and organization. The preferred material to protect the clot in Guided Bone Regeneration is the titanium foil, as it can be shaped according to the bone defect. Furthermore, its exposition in the oral cavity does not impair the procedure. We report on five clinical cases in order to explain the management of blood clots in combination with titanium foil barriers in different clinical settings. Besides being the best choice to protect the clot, the titanium foil represents an excellent barrier that is useful in GBR due to its biocompatibility, handling, and mechanical strength properties. The clot alone is the best natural scaffold to obtain the ideal bone quality and avoid the persistence of not-resorbed granules of filler materials in the newly regenerated bone. Even though clot contraction still needs to be improved, as it impacts the volume of the regenerated bone, future studies in GBR should be inspired by the clot and its fundamental properties.

## 1. Introduction

In the last five years, scientific articles about bone regeneration have demonstrated how surgical techniques and materials have constantly been developing. This aspect, associated with the evolution of dedicated biomaterials, offers multiple surgical approaches and opens new scenarios in patient selection and management [1,2,3,4,5,6,7,8,9].

Thanks to tissue engineering and regenerative medicine, the development of bio-matrixes and scaffolds is increasing with the aim to induce tissue regeneration through the stimulation and the enhanced production of adhesion molecules and growth factors, creating an optimal extracellular environment.

The ideal regenerative materials should be applied *in situ*, avoiding pathological effects on the adjacent tissues and they should promote the enzymatic processes that lead to remodeling and substitution of the matrix as in physiological extracellular matrix (ECM) [10]. Indeed, tissue engineering aims at the development of grafts made by functional tissue capable of regenerating and substituting the physiological tissues, following a biomimetic approach.

In tissue engineering, the driving principle is to provide the right environment in order to steer cell differentiation with the desired phenotype at the right time and place [11]. To achieve these results, materials and methods are fundamental. This regenerative philosophy is discordant with the use of heterologous biomaterials that are not completely resorbed or that take a long time for integration into the host tissue [12,13,14].

Even if autologous bone transplantation is currently considered the gold standard in clinical practice, this approach presents variable reabsorption timing [15,16,17,18,19] and the procedure requires surgical techniques at high risk of complications [20]. Therefore, starting from Murray’s intuition about the potential of clots as a natural scaffold in bone regeneration [21,22,23] and after the considerations based on his analysis, supporting evidence about the utilization of the blood clot in Guided Bone Regeneration (GBR) is actually required [24,25,26].

## 2. The Blood Clot

Murray demonstrated how, in a bone defect, it is sufficient to maintain the defect space to lead a spontaneous bone regeneration in a shorter time than a bone graft. Due to this fundamental observation, we analyze clot transformation starting from clot formation [21].

Clot formation is the first step in the wound healing process that immediately follows such traumatic events as dental extraction or bone fracture [27,28]. Kolar et al. [28] focused their studies on the immune response in the early stage of fracture healing while de Sousa Gomes et al. [27] described the clot arrangement and evolution in the post-extractive alveolar bone. For these reasons, clot should be considered a biomaterial containing all the requested information for physiological bone healing [27].

A histomorphometric study on primates underlined the events succeeding in the alveolar bone between 4 and 180 days following the dental extraction [29].

Even if different tissues have different growing times, clot organization, driven by peripheral information, is sufficient for bone differentiation as in natural GBR.

Melcher et al. [22], created a shield to isolate the bone defect and observed the proliferation of neo-generated bone grown over the edges of natural bone. Even in this case, it is possible to assert that bone regeneration started spontaneously from clot organization.

Due to the role of the blood clot in the early stages of the healing process and due to a clot being considered a concentrate of blood products, during these last years, many studies were focused on blood concentrates such as platelet-rich plasma (PRP), platelet-rich fibrin (PRF) and platelet-rich growth factor (PRGF) [30,31]. The behavior of blood derivates is different from the whole blood. There is no evidence sustaining that separation of blood concentrates from erythrocytes causes morphologic alterations in surgical sites [32,33,34,35]. In fact, erythrocytes play a pivotal role in clot formation, first of all in the proliferation of homogeneous fibrin networks. Following these observations, Wang et al. identified the clot as the best natural scaffold to fulfill bone defects, thus avoiding toxic reactions usually induced by a high concentration of growth factors [23]. In clinical practice, the use of blood products is the best option to achieve soft-tissue healing [27]. To corroborate this statement, it is interesting to observe the behavior of clots in the sinus lift. Lundgreen et al. [24] reported the new bone formation in sinus floor elevation following spontaneous healing, without any filler. Following this observation, implant techniques involving sinus lift, without the use of biomaterials, were developed [36,37,38].

Chipaila et al. [26] measured, for six months, the implant stability quotient (ISQ) after performing a sinus lift with simultaneous insertion of implants using equine collagen sponge as filling material. They noted that the ISQ was stabilized between the fourth and sixth months. Therefore, a biologically active bone was noted around the implants.

Lambert et al. [39] analyzing different methods for subantral regeneration in rabbits, compared protocols involving blood clot, autologous bone and bovine hydroxyapatite (BHA) as a filler.

All three fillers retained the space for bone formation and guaranteed space maintenance, but only blood clots caused a loss in volume after 5 weeks from surgery.

It is on the basis of these considerations that we believe that the use of the clot is a good natural scaffold but that it needs adequate protection to prevent reabsorption: the titanium foil.

## 3. Clot Management and Titanium Foil: Biological Principles

In addition to sinus lift protocols, other studies consider the possibility of using the clot as the main filling scaffold in GBR procedures [40,41,42,43].

Standard titan foils could be shaped during the surgical procedures or pre-shaped thanks to the CAD-CAM technology that gives a model of the jawbones.

As suggested by Elgalli et al., stiffness and plasticity are the main features of these barriers [3,25,44,45,46,47,48,49].

Membrane exposition, usually considered as a surgical complication, [8,50,51,52] is in this case considered as an intentional procedure above all in socket preservation and GBR [53,54,55,56].

In the systematic review of Roca-Millan et al., titanium foils demonstrated a higher tolerability in patients compared to non-absorbable membranes and titanium grids [57].

The rationale beyond the use of titanium is clot protection. Tamura et al. [58] implanted titanium barriers in the rabbits’ calvaria without filling materials, leaving them exposed. The authors observed the formation of new lamellar and trabecular bone with bone marrow having grown inside the barrier [58].

In order to evaluate the importance of the occlusive effect of the titanium barrier, Yamada et al. [59] applied two titanium barriers, one occlusive and one perforated, indicating that within the occlusive capsules, a predictable mineralized bone matures in the spaces beyond the skeletal envelope [59].

In another study on rabbits, Ezirganlı et al. [60] used autologous blood, deproteinized bovine bone (DBBG) and biphasic calcium phosphate bio-ceramic graft material as fillers in three different controls. Even though no significant differences between fillers were reported, the regeneration of bone tissue was obtained only using clot as a filler; on the contrary, the regenerated tissue obtained using other fillers had inclusions of graft material. Moreover, the authors noticed an improved osteoclastic activity in BCP rather than DBBG [60].

Van Steenberghe et al. [25] conducted their clinical study on patients with severe atrophy of the upper jaw. They were treated with customized titanium occlusive barriers filled with the clot. Follow-up was 12 and 18 months and after the removal of the barriers it was possible to observe large volumes of regenerated bone allowing the implantation of 33 implants. The authors concluded that this innovative technique was highly predictable in the regeneration of large bone defects and was to consider the new gold standard in jaw rehabilitation with implants.

We report on 5 heterogeneous clinical cases to support and demonstrate the previously described characteristics of titan foil and clot. The cases are different from each other because they emphasize the different uses and properties of the titanium barriers and the clot, in different clinical situations and settings.

## 4. Case Series

### 4.1. Clinical Case n.1.

**Figure 1:**
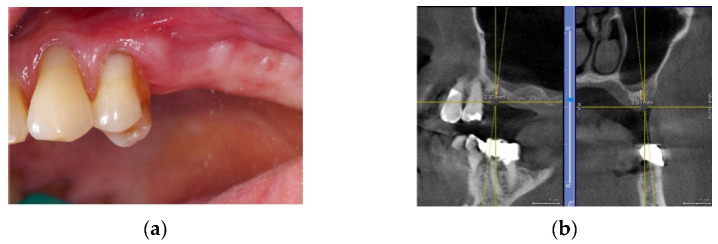
(**a**,**b**): First quadrant clinical aspect and TAC X-ray image.

This 45-year-old male patient was scheduled for prosthetic rehabilitation of the posterior maxillary area. After a radiological check, it was decided to proceed with a lateral sinus lift approach (Figure 1a,b).

**Figure 2:**
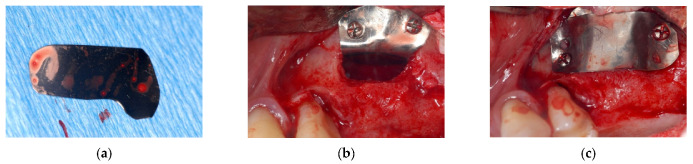
(**a**) shaped foil; (**b**) foil fixed to the bone window; (**c**) bone surgical site isolation.

A full-thickness flap was incised, and a bone window was made. A 25 × 35 mm foil was “L” shaped and applied in the window bone to allow its short side to be fixed on the vestibular bone and the long side overturned inside the sinus to maintain the created volume (Figure 2a,b).

The space between the sinus floor and the sinus membrane was filled with blood clot and a second foil was shaped and fixed to vestibular bone in order to separate the cavity from the surrounding soft tissues (Figure 2c).

**Figure 3:**
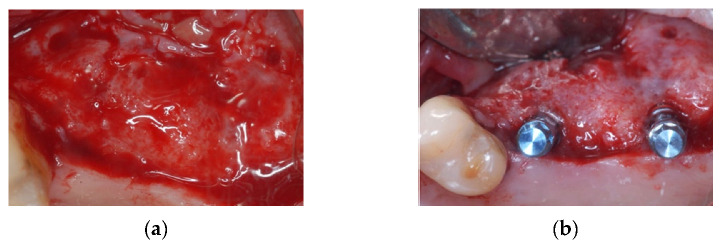
(**a**) newly formed bone tissue; (**b**) implant placement.

Six months later, a full-thickness vestibular flap was made (Figure 3a) to remove the foil barriers and to proceed with the implant insertion (Figure 3b).

**Figure 4:**
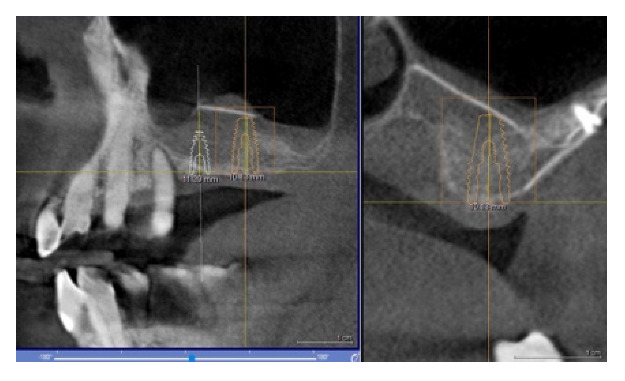
Foil radiographic checks: in evidence the shape of the foil.

The pre-implant CT showed (Figure 4) the new bone volume obtained with the procedure, which was sufficient to allow the implant insertion.

**Figure 5:**
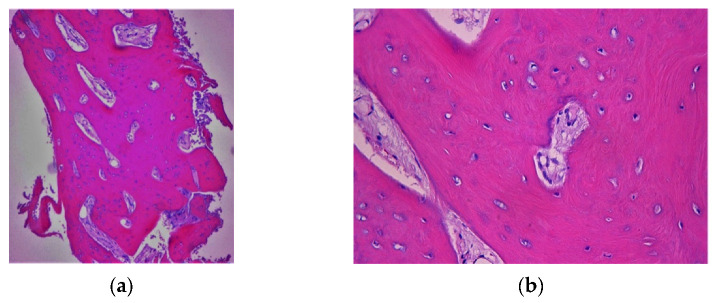
(**a**,**b**) histological examination which confirms the presence of only newly formed bone tissue.

The histological examination of the regenerated bone evidenced the presence of newly generated bone tissue (Figure 5).

In this case, it is shown how titanium foil could be employed in sinus lift thanks to its ability to maintain the space filled with blood clot.

### 4.2. Clinical Case n.2.

**Figure 6:**
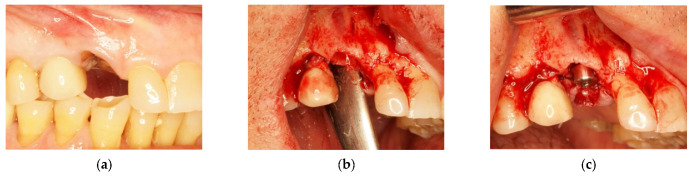
(**a**) Edentulous tooth site 13; (**b**) evaluation of the alveolus; (**c**) implant insertion.

**Figure 7:**
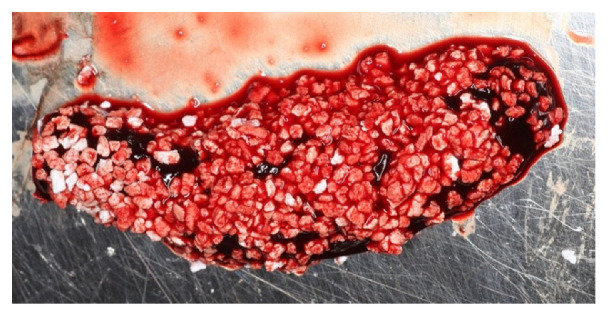
Peripheral blood clot with beta-tricalcium phosphate particles in granules.

In order to rehabilitate the missing 1.3, an immediate post-extractive implant for tooth 1.3 was planned (Figure 6a–c). Blood clot was collected directly from patients (peripheral venous blood) and placed in a 5 mL vial. When the clot was thick enough due to coagulation, it was enriched with beta-tricalcium phosphate by rolling it on the surface of tricalcium granules: 1 g beta-tricalcium phosphate for every 5 mL of clot (Figure 7). Beta-tricalcium phosphate was chosen to stabilize the clot volume and for its capacity to be reabsorbed [61,62,63].

**Figure 8:**
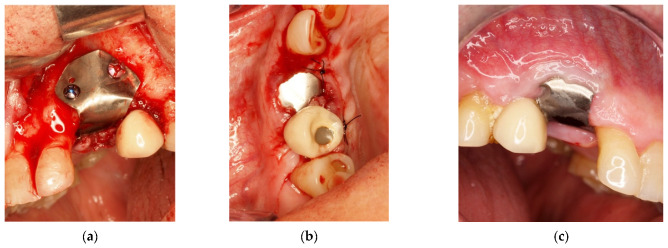
(**a**) Protection of the alveolus with titanium foil; (**b**) titanium foil; (**c**) follow-up at 6 months.

After the implant insertion, the enriched clot was put in the alveolar gap and protected by a shaped intra-operatory titanium foil, fixed with screws on both buccal and palatal walls. It sutured, leaving the foil exposed (Figure 8a,b).

Six months after surgery, the high quality and stability of soft tissues was observed (Figure 8c).

**Figure 9:**
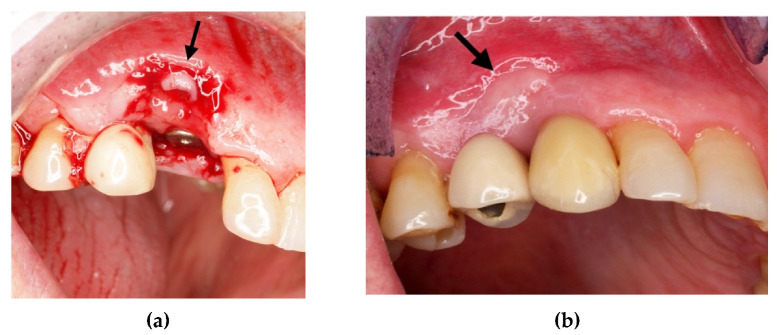
(**a**) Flap of keratinized mucosa indicated by the arrow; (**b**) finalization with a prosthetic crown.

The titanium barrier was removed and the growth of new bone tissue, even if not completely mature, was observed (Figure 9a). After a further 6 months, we noted how pre-existing keratinized gingiva of keratinized mucosa indicated by the arrow in Figure 9a, contributed to the formation of the adherent gingiva band as shown in Figure 9b.

### 4.3. Clinical Case n.3.

**Figure 10:**
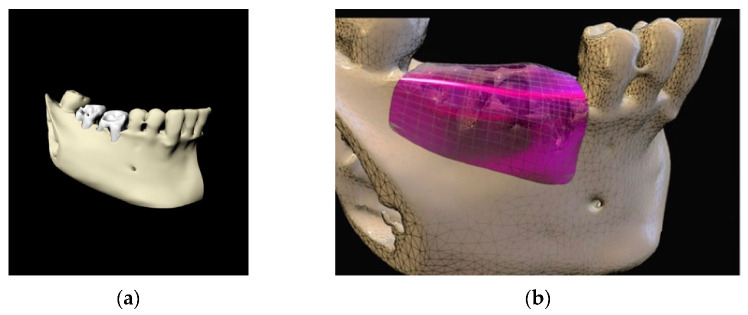
(**a**) 3D model of the mandible; (**b**) Titanium foil digital design.

In this case, we observed how modern 3D reconstruction techniques can be a good support in regenerative surgery. In fact, starting from dicom files format, obtained with cone-beam computed tomography (CBCT) (Osteophoenix, Erandio-Vizcaya-Spain) it was possible to simulate “ex-ante” the surgical procedure. In addition to the reconstruction of the mandible (Figure 10a), the extraction of molars 4.6 and 4.7 was also simulated.

A titanium foil barrier was preventively designed and prepared in order to shorten surgical time (Figure 10b). In fact, the great advantage in cases like this is that the device does not need to be shaped during the surgical session.

**Figure 11:**
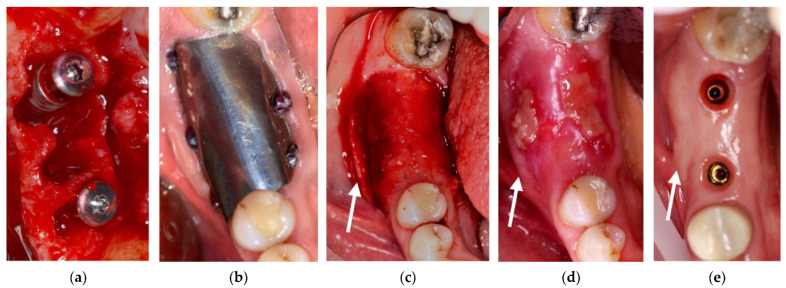
(**a**) Avulsion of the teeth and simultaneous insertion of the implants; (**b**) Use of the enriched clot and cover with the foil blocked with the screws; (**c**) Removal of the foil at six months; (**d**) Maturation of the tissues after one week from the withdrawal of the foil; (**e**) pre-prosthetic situation of implants and mucosa.

**Figure 12:**
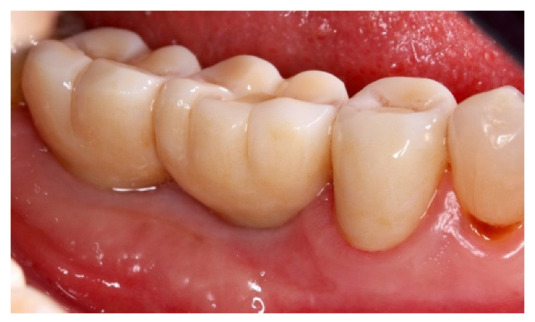
Prosthetic finalization of the case and mucose quality.

The surgery protocol was the same as described in the previous case (Figure 11a–e). The extraction of teeth and the simultaneous insertion of the implants was performed in the same session (Figure 11a). The enriched clot (1 g beta-tricalcium phosphate for every 5 mL of clot) was protected by the shaped foil fixed with screws (Figure 11b) and its successive removal was at six months (Figure 11c). A further six months were necessary for soft tissue maturation (Figure 11d,e). In this case, the flap of keratinized mucosa, indicated by the white arrow in Figure 11c–e, contributed to the stability of the keratinized mucosa. At the follow-up, the gingival tissue was adequately matured, avoiding further graft of soft tissue (Figure 12).

### 4.4. Clinical Case n.4.

**Figure 13:**
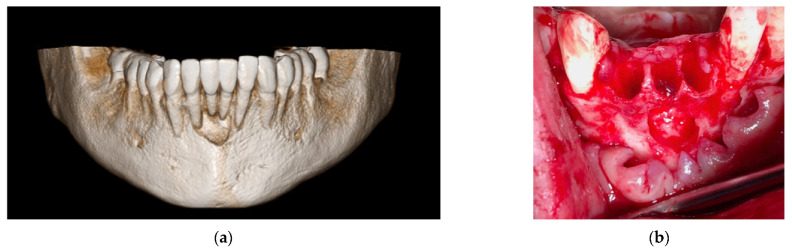
(**a**) Cystic lesion between lower incisors; (**b**) full-thickness flap, extraction of the three incisors and removal of the cyst.

In this case, a cystic lesion involving three lower incisors was treated (Figure 13a).

**Figure 14:**
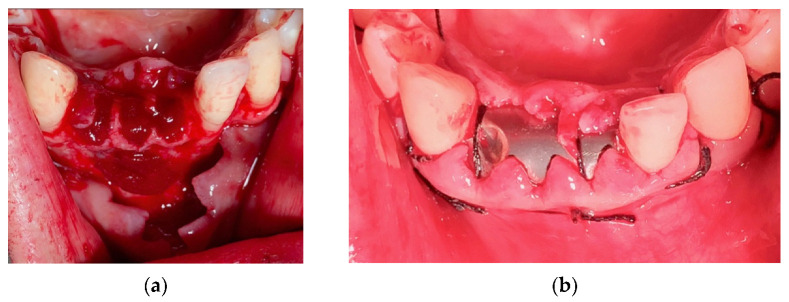
(**a**) Extraction site filled with collagen sponges; (**b**) titanium foil and suture.

Following a full-thickness flap, three incisors (4.2, 4.1 and 3.1) were extracted (Figure 13b). After a careful curettage of the cyst cavity, collagen sponges were grafted in the cavity. The collagen sponges were wet with surgical site blood (Figure 14a). A titanium foil (Figure 14b) was shaped and positioned and the flap sutured.

**Figure 15:**
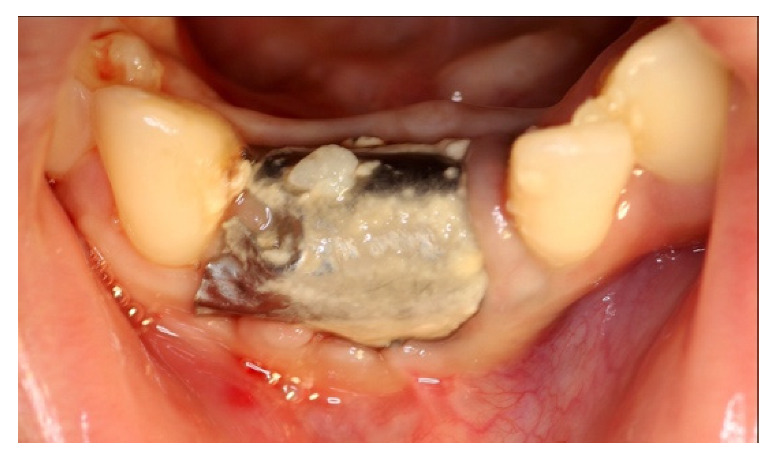
Accumulation of plaque on the foil.

At the follow-up, a significant plaque accumulation on the foil surface was observed; nevertheless, there were no significant signs of soft tissue distress (Figure 15).

**Figure 16:**
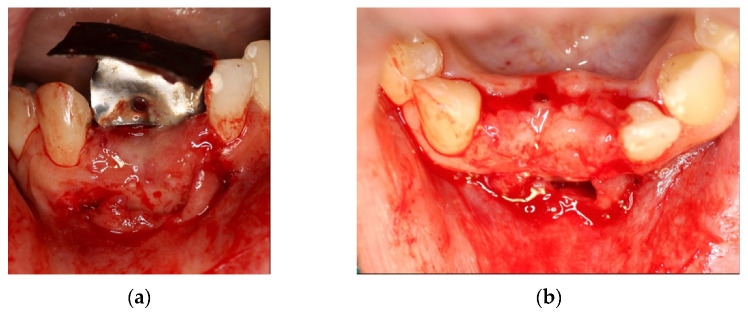
(**a**) Removal of titanium foil after 4 months; (**b**) osteoid tissue with hard but still elastic consistency covered by not-epithelized mucosa.

**Figure 17:**
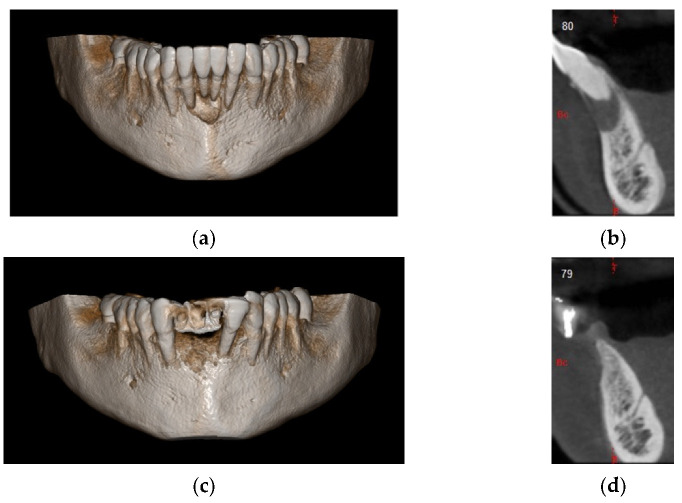
(**a**) Initial 3D situation; (**b**) CBCT of the initial situation; (**c**) Final CBCT post 3D regeneration after 4 months; (**d**) X-ray of the situation post regeneration after 4 months.

4 months after the surgery, the barrier was withdrawn and, behind it, growth of elastic and thick osteoid tissue was observed (Figure 16a,b). After further 4 months, a CBCT was performed, and the amount of regenerated bone was observed (Figure 17a–d).

**Figure 18:**
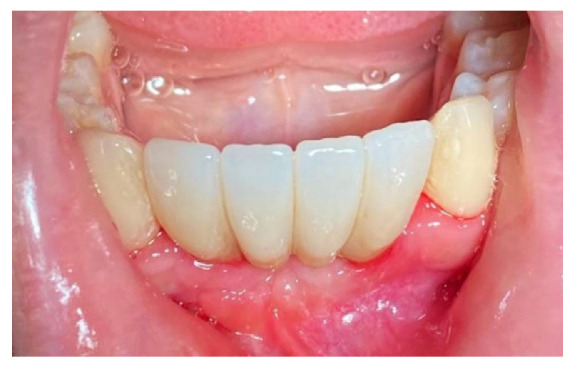
Prosthetic finalization of the case and mucose quality.

The patient was prosthetically rehabilitated with additional implants positioned in 4.2 and 3.2 (post-extractive). In this case, we also noted how soft tissues surrounding the regenerated site were mature and stable (Figure 18).

### 4.5. Clinical Case n.5.

In this case, we report a procedure based on biological principles reported by Polimeni et al. [64] and Komman and Robertson about the regenerative properties of the periodontium [65] and surgical procedures, are factors associated to the correct management of the periodontal defects. Wikesjö et al. [66] reported that the extension of the periodontal regenerated area was better in sites treated by occlusive membranes rather than the porous one. Moreover, Luo et al. [67] in an experimental study, reported better outcomes in premolars furcation healing treated with blood clot pointed out the larger regenerative potential of the clot rather than bone-chip.

**Figure 19:**
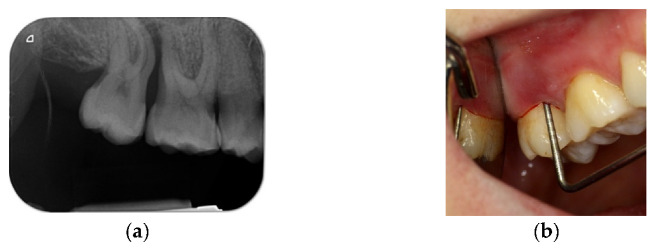
(**a**) Periapical radiograph of teeth 1.7, 1.6, 1.5; (**b**) important probing periodontal mesial root of 1.7.

In this case of chronic periodontal abscess between teeth 1.7 and 1.6, there was an important buccal and palatal periodontal pocket of 9 mm with a large resorption of the interdental bone septum. It was decided to extract the tooth 1.7 because of its poor prognosis (Figure 19a,b).

**Figure 20:**
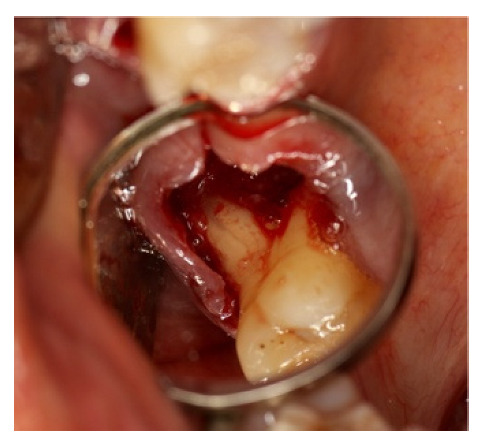
Furcation lesion between the distal buccal root and the palatal tooth 1.6 after 1.7 extraction.

A large amount of infected granulation tissue was present in furcation space, between the distal-buccal and palatal root (Figure 20).

The alveolar cavity and roots were curetted and cleaned with a solution of physiological water and hydrogen peroxide 1:1 and collagen sponges, completely wetted by the blood from the surgical site, were applied and followed by the protective positioning of a shaped foil.

**Figure 21:**
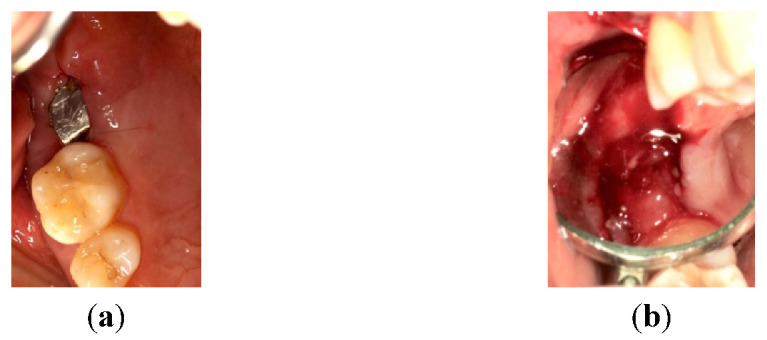
(**a**) Removal of sutures after 3 weeks; (**b**) extensive healing tissue is observed which has completely covered the previously exposed roots.

After three months, the foil was removed (Figure 21a,b) and the roots surface previously exposed, appeared covered by bone.

**Figure 22:**
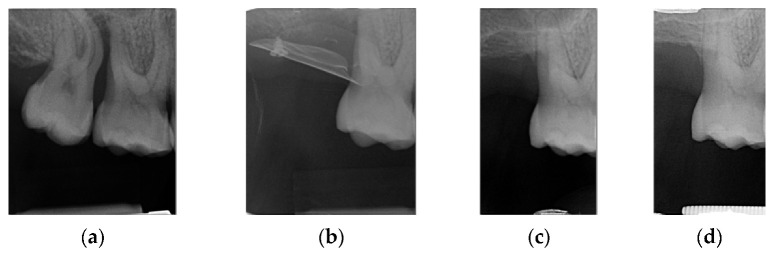
(**a**) Initial periapical radiograph; (**b**) periapical radiograph after foil placement; (**c**) periapical radio-graph after 6 months; (**d**) periapical radiograph after one year.

A Comparative radiological analysis before and after the surgical procedure, showed an increase in the distal bone peak. The gained bone was also maintained after 6 months of follow-up (Figure 22c). After one year (Figure 22d), the distal bone peak was comparable in height with the vestibular one. 

This is a further demonstration of how clot alone (a natural scaffold) induces the best regenerative abilities of the residual bone.

## 5. Discussion

In our case series, we report different clinical situations connected by the common thread of the blood clot. Starting from biological considerations about using blood clots as a scaffold we proceeded to make suggestions for its protection through the use of titanium foil as a barrier.

We evidenced how titanium foil is a biomaterial that is easy to handle, tolerated by the tissues and respectful of the biological processes that guarantee the healing and the formation of new bone tissue. Its association with the clot stabilized by beta-tricalcium phosphate, makes the GBR procedures more predictable.

Moreover, the described biomaterials, have allowed in different and heterogeneous clinical situations, to customize the treatment, according to the anatomical and surgical needs.

Several studies evaluated the differences between occlusive barriers and resorbable membranes and indicated the different use according to the characteristics of each case [68]. In other studies, attention was paid to the bioactive role of the barrier. Other studies stated that the barrier guarantees the maintenance of space, allowing selective permeability [3,5]. In this way the two compartments (bone defect and soft tissue), separated by the barrier, act in synergy. It is certainly interesting that this will happen in the future, but we are still far from understanding the mechanisms of this interaction as, for example, in the case of the role of periostin [69,70].

As stated in Cucchi et al. [8], in controlled and randomized clinical studies, there are no significant differences between d-PTFE membrane and titanium grids covered by collagen membrane, neither in regenerated bone volume nor in clinical complications. Soldatos et al. [71] supported the theory in which both kinds of membranes have limitations that affect the clinical outcomes. In fact, the most helpful material in exposure is the titanium foil, as reported by Roca-Milan et al. [57]. The use of titanium barriers is increasing in clinical practice [48,49]. The biological behavior of the mucosa around the surfaces of dental implants is well known. The soft tissues tend to create attached epithelium with the titanium surface, in an area of about 2 mm apically from the soft tissue edge [72]. For these reasons it is possible to use a highly bio-compatible and inert material that can avoid mucosal alterations in intentional and accidental exposition. In fact, by following an appropriate hygiene protocol, it is possible to control plaque formation [73].

Titanium barriers are the best solution, due to versatility and manageable features. These features assure shorter and better surgery and wound healing practices.

In a comparative study between bone graft and GBR performed with titanium foils and clot alone, Molly et al. [45] analyzed the gained bone volume obtained with two different techniques: bone grafts and GBR made with Ti barriers with the clot as a filler. Dental implants were subsequently placed in the same sites. The bone volume obtained with the barriers was lower, however the resorption of peri-implant marginal bone proved to be more stable over time than the implants inserted into the grafts.

The two main critical issues in the use of clot are the scarce dimensional stability resulting from contractions, and the interaction between platelets and the surrounding environment [74].

Platelet contractions re-shape fibrin networks by improving the density and stiffness of the blood clot. Fundamentally, platelets compact the fibrin network (i.e., densify the fibrin network by pulling the fibers transversely to their longitudinal axes). New observations about platelet contraction in clot showed potential implications in the biological application of cell motility [75].

Since platelets have the property of mechanosensing (the ability to recognize substrates with variable stiffness and activate accordingly) it is possible to exploit this property to modulate the density and stiffness of the clot itself [76].

These claims justify why Molly et al. found less bone volume at the end of their experiment. Based on these claims, we can assume how important it is to use clot in association with a filler that, through its biological activity and without interfering with the clot, can confer a main support function expressing those inherent properties peculiar to the clot-scaffold. In our opinion, beta-tricalcium phosphate is the best candidate as filler to associate with the blood clot.

## 6. Conclusions

Titanium foil represents an excellent barrier that is useful in GBR, and it is also the best choice to protect the clot, due to its biocompatibility, handling and mechanical strength properties.

Furthermore, if the clot alone induces a higher quality bone at the expense of bone volume, we hope that tissue engineering will discover a suitable material to support the clot and/or act as the clot. We now think that the clot is the model to inspire future studies.

## Data Availability

The data presented in this study are available on request from Lucio Milillo (lmilillo1@tin.it). The data are not publicly available due to Italian law about personal privacy (D. lgs. 30 June 2003, n. 196).
